# Understanding the influence of online grocery shopping on consumers’ choice of products and dietary balance: a qualitative study in France

**DOI:** 10.1017/S1368980025000266

**Published:** 2025-05-17

**Authors:** Olivier Droulers, Sophie Lacoste-Badie

**Affiliations:** 1 University of Rennes, NeuroLAB CREM (UMR 6211), F-35000 Rennes, France; 2 University of Lille, LUMEN (ULR 4999), F-59000 Lille, France

**Keywords:** Online grocery shopping, Balanced diet, Consumer decision-making, Food consumption

## Abstract

**Objective:**

To explore the impact of online food shopping in France on the selection of products purchased and its potential impact on shoppers’ dietary balance.

**Design:**

A qualitative study involving in-depth semi-structured individual interviews. The interviews were recorded, transcribed verbatim and analysed through a reflexive thematic analysis approach.

**Setting:**

France

**Participants:**

Thirty-four male and female respondents aged between 21 and 61 years old, residing in various regions of France, including urban, suburban and rural areas, with diverse profiles in terms of gender, age, location and number of children under 18.

**Results:**

Five key themes were identified as influencing decision-making with regard to the products purchased, namely ‘less choice, especially for fresh produce’, ‘sense of security in buying the same products’, ‘convenience of online shopping through time-saving and product recommendation lists’, ‘avoiding unplanned purchases’ and ‘less fresh produce purchased, sometimes replaced by more processed items’. In turn, all of these factors potentially have an impact on the diet of online shoppers.

**Conclusions:**

With grocery e-commerce penetration expected to double in the next 5 years, the study underscores the consequences of online shopping on consumers’ dietary balance. The findings have practical implications for online food retailers, inciting them to develop solutions that would encourage e-grocery shoppers to buy more fresh produce and sample a more varied diet. Additionally, they highlight the importance of monitoring the influence of technology on the consumer buying process, particularly with regard to food.

In 2023, e-commerce accounted for almost 19 % of retail sales worldwide and this number is expected to grow by 25 % by 2027^(1)^. Within this large expanding market, the main driver of e-commerce has long been the purchase of non-food items such as clothes, electronics and footwear. However, online grocery shopping (OGS) has grown rapidly since the COVID-19 pandemic^(2)^. OGS is defined as ‘the purchase of food or personal use items via a food retail company’s Internet-based portal or application with delivery at the consumers’ home or designation or in-store pickup’^(3)^. Historically under-penetrated relative to other e-commerce categories^(4)^, OGS has gained significant traction in recent years. According to Kantar Winning Omnichannel^(5)^, the global share of the e-commerce grocery market amounted to 7·2 % in 2021, compared to 6·3 % in 2020 and 4·8 % in 2019. With new consumers reporting satisfaction with the system as retailers continue to improve their offers and services, the size of the global online grocery market is predicted to reach USD 2827·63 billion by 2032, up from 365·74 billion in 2022^(6)^.

The emergence of such disruptive innovation in the retail sector has sparked both academic and commercial interest and has been examined from various angles. One area of academic interest concerns the drivers and barriers behind consumers’ willingness to shop for groceries online^(7,8)^, while another pertains to the impact of situational factors^(9)^. Individual factors have also been explored, such as age-related differences in behaviour and attitudes towards online grocery shopping^(10)^, personality traits^(11)^ and socio-demographic factors^(12)^. However, despite a substantial body of research focusing on the antecedents of OGS adoption, our understanding of the consequences of online food shopping is particularly limited, especially regarding its potential impact on consumers’ product purchase decision-making and, consequently, on shoppers’ dietary balance. A balanced diet needs to be both varied – involving the daily consumption of different foods from within the same food group – and diversified, i.e. foods need to be selected from each of the different food groups every day^(13)^. The few studies that have been conducted have resulted in non-convergent conclusions. Some argue that consumers are likely to choose fewer unhealthy alternatives in an online as opposed to an offline food shopping environment^(14)^, while others argue that the absence of physical interaction with products in the online context means that consumers are less likely to purchase perishable items such as fruit and vegetables^(15)^, which are essential for a healthy and balanced diet. To address this gap in the literature, and in response to a recent call^(2)^ to explore the nutritional implications of online grocery shopping, our study seeks to contribute to the OGS literature by investigating the following question:

Does online grocery shopping influence consumers’ decision-making with regard to the products purchased and, potentially, their diet?

## Literature review

Numerous studies, including some conducted during the emergence of OGS in the 1990s^(16,17)^, have attempted to identify factors that incite consumers to shop for food online and those that deter them from doing so. Research shows that participants view the main benefits of shopping for food online through various, broadly functional factors^(7,18)^, such as saving time, being able to place orders regardless of the time of day, easy price comparison, reduced physical effort, avoiding crowds and having to stand in line and reducing impulse buying. The main disadvantages include mistakes with orders and inadequate substitutes, less choice than in brick-and-mortar stores, absence of personal contact, online shopping fees and product quality that cannot be gauged in person^(19,20)^. Various theories and models have been developed to explain the adoption of disruptive innovation, such as the theory of reasoned action^(21,22)^ and the theory of planned behaviour^(23,24)^. These include the technology acceptance model^(25)^ which, given its strong theoretical basis and empirical support, is claimed to be the most influential and widely used to predict the acceptance and usage of various technologies^(26)^. The model suggests that when users are presented with a new technology, two key factors lead people to accept or reject the innovation, namely, perceived usefulness and perceived ease of use^(27)^. Confirming the model’s relevance in the context of Australia^(28)^, Malaysia^(29)^, Thailand^(30)^ and the USA^(31)^, research shows that the perceived usefulness and perceived ease of use of OGS has a positive impact on attitude and/or intention to use, which in turn influences its actual usage.

However, in addition to the characteristics specific to the innovation, situational factors can also affect the decision to adopt online grocery services. Among such factors, life events are an important catalyst in OGS adoption. Two circumstances are frequently identified in the literature^(32)^. The first is having a baby. This event was found to trigger the decision to start shopping for food online in several qualitative studies (e.g. ‘*My sister has just had a baby and she shops online*.’^(33)^; ‘*You don’t have to worry about having the stroller, putting the baby in the car, and then going up and down the aisle*.’^(34)^. The second circumstance is related to temporary conditions as well as more long-term physical limitations (e.g. ‘*Johan had broken his leg […] and it was all a bit too much for me, so I had to find something I didn’t have to take care of anymore*.’^(35)^; ‘*I’m disabled and can’t get out much. So I use my computer to shop from home*.’^(19)^. However, a third major situational factor came to the fore at the end of 2019 with the emergence and rapid spread of a new coronavirus (SARS-CoV-2) across the globe. Strict rules restricting citizens’ freedom of movement, along with social distancing measures imposed by governments to fight the pandemic and the fear of being contaminated while shopping, led consumers to increasingly adopt OGS. Reports suggest that the Covid-19 pandemic advanced Europe’s e-commerce transition by 4–5 years, especially in food retail^(36)^.

Despite the considerable amount of research on the antecedents of OGS adoption, less attention has been paid to the consequences of shopping online for food. One issue in particular remains understudied: the influence of OGS on product selection and its potential impact on shoppers’ dietary balance. Analysing data from a large European retailer that operates both a brick-and-mortar grocery store chain and an online store, Huyghe et al. (2017)^(14)^ argue that shoppers choose relatively fewer ‘vices’ (unhealthy food alternatives) when they shop online compared to offline. The authors identified five ‘vice categories’ with reduced online consumption (candy bars, chocolate, chips, salty snacks, sweets, and chewing gum) and explained this behaviour by the symbolic presentation of products online (as opposed to physical presentation offline) that weakens the products’ vividness. Their decreased vividness reduces consumers’ desire for immediately gratifying products (i.e. ‘vices’), leading them to purchase relatively fewer such products in an online shopping environment compared to an offline one. However, other studies suggest that there is no significant shift in the health value of purchases when comparing online and offline grocery shopping. Evaluating the effects of online grocery shopping on the composition and quality of purchases from grocery scanner data generated from 25 000 households who shop at a traditional brick-and-mortar supermarket that also has an online grocery shopping website, Harris-Lagoudakis (2022)^(37)^ concluded that despite finding small changes in monthly budget allocations across Thrifty Food Plan product categories, summary measures of healthfulness did not show consistent changes upon the introduction of the online shopping service. On the other hand, some studies suggest that concerns about freshness and food safety make consumers less likely to purchase perishable foods online such as fresh fruit, vegetables and meat that are essential for a balanced diet. Based on ethnographic case studies, Elms et al. (2016)^(38)^ observed that some consumers tend not to buy fresh or perishable items online due to the limitations of multisensory assessments that involve touching, smelling, examining or even tasting the products, which are not possible in an online setting^(15)^. Interestingly, in a study designed to quantify consumer motivations and benefits, the authors showed that nutritional goals and healthy eating play a minor role in consumers’ choice to engage in OGS^(7)^. However, while most consumers do not shop online specifically to improve their diet, it does not mean that OGS has no effect on their product purchase decision-making and, consequently, on their diet. Recently, Bennett et al.^(39)^ conducted a systematic scoping review on the digital food retail environment’s potential influence on health. The review showed that numerous studies focus on specific populations, such as adults with limited access to transportation or overweight participants. Some studies have reported that customers claim they purchase fewer unhealthy foods and beverages when shopping online compared to in-store shopping. However, other studies present mixed results. For instance, Gorin et al.^(40)^ found no significant difference in weight loss between the intervention group instructed to order groceries online instead of shopping in-store and the control groups, while Sacks et al.^(41)^ demonstrated that providing more detailed nutritional information on a supermarket website did not lead to significant changes in real-world online sales. Additionally, some customers reported that shopping online reduced their ability to choose healthy foods and beverages as it was more difficult to compare products. In another scoping review, Jilcott Pitts et al.^(42)^ identified the potential promises and pitfalls presented by OGS in relation to food and beverage purchases. We compare their findings with those of current research in the discussion section. Given the limited and inconclusive nature of existing research, this study attempts to deepen our understanding of how shopping for groceries online influences decision-making with regard to the products purchased and consumers’ subsequent diet.

## Method

We adopted a qualitative approach since it is well-suited to investigating and developing an in-depth understanding of a currently under-explored issue^(43)^.

### Data collection

In-depth, semi-structured individual interviews were conducted with thirty-four male and female respondents aged between 21 and 61 years. They come from across France and live in towns, the suburbs or the countryside. The profiles of the thirty-four respondents vary in terms of gender, age, location and number of children under 18. Participant inclusion criteria consisted of doing part or all of their food shopping in a digital environment (website or mobile app). The interviews were divided between the two researchers, with each researcher responsible for transcribing their own interviews. Respondents were interviewed via an audio-visual application (Zoom). Data saturation became evident after thirty interviews. However, four more interviews were conducted to confirm that there were no additional or new concepts^(44)^. All the interviews were recorded, transcribed verbatim and de-identified before analysis. The respondents received a gift card of €25 for their participation in the study.

Each interview began by thanking the respondent for taking part in the study. The researcher then gave a general outline of the interview topic and informed the respondents that the conversations would be recorded and that their input would remain confidential, explaining that each person would be given a code number so their name would not be used in any report. After this, the researcher collected the respondents’ consent to participate in the research. To introduce the research topic gradually, the first part of the interview guide addressed questions not directly related to the topic, such as ‘when did you begin shopping online for groceries?’, ‘do you always use the same e-retailer?’ and ‘was there a triggering factor to shopping online for groceries?’. The second part of the interview guide was structured around themes and potential prompts that could affect the online consumer buying process: (a) the share of online and offline purchases, (b) the different places where they can shop for food, (c) online and offline product offers, (d) how the participant uses a website or app to shop, (e) the use of product recommendation lists proposed by the website or app, (f) planned and unplanned online and offline purchases and (g) changes in diet both in terms of quantity (more of this, less of that) and quality (trying new products, excluding certain products) (see Appendix A). The interviewer did not have to do much prompting since most of the participants seemed to find the topic both interesting and easy to discuss.

### Data analysis

The data were examined using reflexive thematic analysis (RTA)^(45,46)^. RTA is a method used to systematically identify, analyse and report patterns (themes) within data. According to Braun and Clarke^(47)^, themes are conceptualised as ‘patterns of shared meaning, united by a central concept or idea’ created from codes and through the researcher’s active engagement with the dataset, rather than emerging from the data as if they existed within it prior to analysis. The analysis was conducted in accordance with the six phases of thematic analysis^(45)^ – becoming familiar with the data, generating initial codes, searching for themes, reviewing themes, defining and naming themes and producing the report, keeping in mind that it requires a continual back-and-forth between the whole dataset, examination of the coded extracts from the data, and analysis of the data produced. The interviews were read multiple times by the two authors to become familiar with the data and to gain a deeper understanding of the content. The authors decided not to divide the coding task but to collaboratively code the data extracts from all the interviews manually. This approach precludes the use of inter-coder agreement, which, according to Braun and Clarke^(47)^, does not threaten the validity of the research. Throughout the coding process, a reflective diary was kept to document ongoing reflections on the findings. Emerging insights were gradually refined and evaluated by drawing on data from an increasing number of interviews. Themes were developed from and through the coding process, leading to five themes reflecting the interview contents.

## Results

Table 1 provides a demographic overview of the study participants. Five principal themes were identified from the data analysis.

**Table 1:** Demographics

	%	*n*
Total participants	100	34
Gender		
Female	76·5	26
Male	23·5	8
Age		
Up to 29	26·5	9
30–49	53	18
50+	20·5	7
Location		
Urban	32·5	11
Suburban	44	15
Rural	23·5	8
Number of children under 18		
0	50	17
1	20·5	7
2	20·5	7
3+	9	3

### Less choice, especially for fresh produce

A large majority of participants stated that when shopping online for groceries, there is less choice across all categories than in traditional supermarkets (‘*There’s much less choice*’ participant (P) 16, female, 32 years old, suburban; ‘*There’s far less choice across all product categories.*’ P5, female, 61 years old, urban; ‘*I wish there was more choice online.*’ P8, female, 45 years old, rural). Participants noted that this is especially true for fresh produce which they described as unprocessed or minimally processed produce, such as fruit, vegetables, fish and meat, excluding those that are preserved through industrial processes (‘*There’s less choice online, especially for fruit and vegetables.*’ P3, male, 45 years old, suburban; ‘*There’s no cold meats deli counter, no cheese deli counter.*’ P6, female, 54 years old, suburban; ‘*There should be more choice in the fresh food section.*’ P12, female, 52 years old, suburban). In response to the widely shared observation of a more limited product range in drive-through grocery services, regardless of the category, two impressions emerged. Some participants acknowledged that there are indeed fewer options but, adopting a broader perspective, argued that this did not particularly bother them. For these individuals, the benefits of drive-through shopping far outweigh the drawbacks: ‘*Yes, of course, it can sometimes be inconvenient, but I wouldn’t go back to shopping in-store for anything in the world*’ (P6, female, 54 years old, suburban). Conversely, for others, the lack of choice led to irritation and even frustration: ‘*It annoyed me when I thought, “Oh, I’d like to try this recipe,” but I couldn’t make it because that one ingredient was missing*’ (P24, female, 21 years old, urban). For a few participants, the lack of choice led them to suspect that it might be intentional – part of a strategy by the retailer to influence their purchase decisions: ‘*The drive-through has far fewer options, forcing you to buy certain products. I wonder if they do it on purpose to make us buy specific items*’ (P4, male, 38 years old, urban).

### Sense of security in buying the same products

According to several participants, not being able to see the product in person makes them more hesitant about trying something new (‘*When I shop online, I only trust the stuff I’m used to, so that limits my options.*’ P19, female, 34 years old, rural). They feel more confident ordering products they already know, in other words, things they have bought in the past (‘*We’re used to certain products and maybe we don’t actually change anything because we’re afraid of being disappointed.*’ P17, female, 50 years old, rural). Some participants grew very wary after encountering relatively common issues with weight or quantity (‘*Sometimes you may have a bad surprise because you read the information wrong. I remember my mother ordering 5 bunches of bananas when she just wanted 5 bananas. So we tend to buy the same products because we know that there won’t be any mistakes.*’ P29, female, 22 years old, urban; ‘*I thought I’d ordered a 150g butter pack and I ended up with a 500g pack.*’ P10, male, 32 years old, rural). However, once their initial disappointment or frustration caused by the difference between the product they expected and the one they actually received has subsided, most participants do not appear to be significantly affected by these errors in the long term. During the interviews, none of them suggested that they might abandon online shopping in the medium or long term following such an experience. They either suggested that they adapted to this constraint by purchasing products they already knew – i.e. products they had bought before – while others gave the impression of feeling reassured by making repeat purchases. Moreover, they tended to place responsibility for any issues they experienced on themselves, perceiving it as their own mistake rather than placing blame on the retailer.

### Convenience of online shopping through time-saving and product recommendation lists

Participants stressed that one of the main reasons for shopping online was to save time (‘*It takes far less time.*’ P20 female, 57 years old, suburban; ‘*It saves all the time wasted wandering around the store.*’ P21 female, 30 years old, suburban; ‘*I find that going to a store with all the people there is a waste of time.*’ P24 female, 21 years old, urban). Participant 19 (female, 34 years old, rural) said that she used to spend all her Saturday mornings doing in-store grocery shopping. Now she shops online during her lunch break at work using her smartphone and just has to pick up the order at the end of the day. Most of the participants said that they used a list generated by the online retailer to save time. This could be the list of products from the last order or a ‘favorites’ list which reiterates the most frequently purchased products (‘*I often use the recorded list of products.*’ P2, female, 34 years old, rural; ‘*I follow the automatic list a lot, it’s the one with my favorite products that I choose most often.*’ P6, female, 54 years old, suburban). Participants reported that the product recommendation lists tend to incite them to frequently buy the same products (‘*With the pre-recorded list, I tend to buy the same products more often. Before, when I went to the shops, I used to vary my purchases more.*’ P11, female, 52 years old, urban; ‘*As we tend to use the same order as before, the basics are more or less always the same.*’ P18, female, 35 years old, suburban; ‘*As we buy virtually the same things every week, there’s a lot less variety. We get variety again when we decide to invite some people round and then we go shopping in the stores.*’ P1, female, 39 years old, suburban; ‘*It’s usually the same products with click and collect. We have a short list of products and we regularly dip into the short list without necessarily adding anything else.*’ P4, male, 38 years old, urban; ‘*Sometimes I don’t add anything new to my list for two months at a time.*’ P19, female, 34 years old, rural).

### Avoiding unplanned purchases

Participants stated that online grocery shopping gives them more control over their budget as they make fewer unplanned purchases when shopping online (“*If I go to a store, I know that I’ll definitely see things that tempt me, and so I’ll suddenly buy more, while I only buy what I planned to online.*” P29, female, 22 years old, urban; “*In the shops, we don’t manage our budget as well because we tend to buy extra things. We keep telling ourselves that we’ve planned to buy just a couple of items, and in the end, we fill half a shopping trolley because when we go along the aisles, we say to ourselves* ‘*Oh yes, I haven’t got that’*.” P23, female, 44 years old, rural). Online respondents have the impression of being less tempted and less exposed to new products (“*Online, I enter the name of the product in the search bar and then I take the brand I know. I don’t look for novelty as much. When you’re in the aisles in a store, it’s true you see things you’ve never tasted before and so you buy them to try them out.*” P22, female, 31 years old, suburban; “*I usually buy the same products online, but in shops I’m more tempted to try new things.*” P25, female, 32 years old, suburban; “*At first we used the click and collect to save time. Afterwards, we realised that we spent less with click and collect than when we went into a shop. We’re not so tempted to buy.”* P1, female, 39 years old, suburban. Prior research shows that unplanned purchases can include both healthy and unhealthy food items^(36)^, echoing what was observed in the data, namely that participants associate unplanned purchases with both unhealthy products (“*In a store, if I see a pizza in an aisle, I tend to take it, whereas I won’t be tempted online.*” P31, female, 29 years old, suburban) and healthy products (”*When I go to a store, I buy more of the things I don’t necessarily buy often. I may see small peppers or dried tomatoes, so I take them.*” P10, male, 32 years old, rural. Avoiding unplanned purchases can thus have a positive or a negative effect on a balanced diet, depending on the type of items purchased.

### Less fresh produce purchased, sometimes replaced by more processed items

Several participants reported having a bad experience when buying fresh products online (‘*I’ve often been disappointed with the quality of the fruit and vegetables bought online.’* P26, male, 36 years old, suburban; ‘*If you really want to buy good quality meat, well you’re not going to find it at the click and collect.*’ P4, male, 38 years old, urban; ‘*With click and collect for fruit and vegetables, we have the impression that there’s less choice, but also that we don’t get the same quality as when we go to a greengrocers.*’ P3, male, 45 years old, suburban; *‘I wasn’t happy with the freshness of the fruit and vegetables.*’ P13, female, 30 years old, suburban; ‘*For very fresh food, like lettuce for instance, I wouldn’t get it from click and collect. I wouldn’t get tomatoes from click and collect either. I tried but I wasn’t happy, I find that they’re often poorly selected.*’ P11, female, 52 years old, urban). Many participants reported that they consequently prefer not to buy fresh produce when they shop online, whether meat (‘*For meat, I go to the butcher.*’ P7, male, 26 years old, suburban; ‘*For meat, I prefer to see what I’m buying, so I go to the butcher.*’ P23, female, 44 years old, rural), fish (‘*I never buy fish online.*’ P19, female, 34 years old, rural), or fruit and vegetables (‘*As I like to choose my own fruit and vegetables, I don’t buy them online.*’ P16, female, 32 years old, suburban). However, participants also reported that since they began shopping online, they have reduced their consumption of fresh produce (‘*Since I started shopping online, I eat less meat or fish than I did before. We haven’t really found a substitute for it.*’ P4, male, 38 years old, urban; ‘*I think that since I’ve been using click and collect to do my shopping, I eat less fresh fruit and vegetables.’* P9, female, 24 years old, urban). More worryingly, in adopting online shopping, some said that they had changed their consumption habits by replacing fresh produce with processed items, such as packaged foods that have longer expiry dates (‘*I don’t buy fresh fish online, so what I take is smoked salmon for instance, which we like a lot, or sometimes tinned fish.*’ P10, male, 32 years old, rural); ‘*Before, when I went to the stores, I often bought (fresh) meat. Since I’ve been shopping online, I’ve been eating less meat as I don’t like it, there’s not much choice, it’s limited. So, I make up for it by buying cold cut deli meat.*’ P17, female, 50 years old, rural). For a significant majority of participants, using the drive-through service is problematic with regard to fresh produce (meat, fish, fresh fruit and vegetables). While the quality of processed and often packaged products is perceived as consistent – delegating the selection of such products to a third party poses no problem, as one can of soda from brand A is identical to another can of soda from the same brand – the situation is different when it comes to fresh products like fruit and vegetables. Participants are aware that within the same category, the quality of fresh produce can vary significantly. As a result, they are far more reluctant to delegate this choice to the person responsible for assembling the drive-through order, with many citing previous negative experiences. To address this issue, some participants report buying fresh products from other sources, generally small specialty shops or open-air markets. However, faced with the handling of two (or more) separate distribution channels – one online for non-perishable goods and another offline for fresh products – some respondents acknowledge that compared with when they shopped in physical stores where they could purchase everything in one place at the same time, their consumption of fresh produce has declined. Others report having replaced fresh products, particularly meat and fish, with processed alternatives.

## Discussion

An increasing number of consumers have turned to online shopping for food in recent years, and experts expect the trend to continue. Previous research has focused primarily on the antecedents of OGS, identifying the factors behind consumers’ adoption of online food shopping. This qualitative inquiry contributes to the existing literature by highlighting the impact of the switch from offline to online food shopping on consumers’ decision-making with respect to the products purchased and, potentially, their diet. To our knowledge, this is the first study conducted on the topic in France, where 90 % of online grocery shoppers utilise ‘click and collect’ services^(48)^ wherein customers retrieve their orders from designated collection points, with preparation of the orders being handled directly by the retailer. This contrasts with countries like the UK^(49)^ and Germany^(50)^, where home deliveries prevail. Several key themes were identified as influencing customers’ purchasing decisions. Respondents said that they regularly buy the same products as choice is more limited with online shopping compared to traditional supermarkets, that they had made mistakes with products selected in the past, and that repeat purchases of familiar products help them to avoid making similar mistakes, confirming previous findings that consumers are more comfortable purchasing products they already know^(51)^. The results also show the significant role of the personalised list of recommended products proposed by grocery e-retailers (e.g. ‘most frequently purchased products’, ‘reorder last order’) on consumer purchases. These lists reflect preferences based on purchase history and are intended to support consumers in their shopping decisions. However, our study data indicate that the use of these lists has an impact on the products purchased. Indeed, many respondents spontaneously acknowledged that the lists incite them to reorder the same things and that their purchases are now less varied than when they used to go to the store. Exacerbated by the use of recommended product lists, lack of variety in the items purchased may constitute a potential problem in terms of a balanced diet. Moreover, many participants reported that they are reluctant to buy fresh produce (e.g. meat, fish, fruit, vegetables) online and that they tend to go to other places such as markets or small local shops to buy such items. However, some said that they do not always take the time to go to specialised shops or markets to buy fresh food and admitted that since they began shopping online, they consume less fresh produce and tend to eat more processed foods (smoked fish, deli meats) and pre-packaged items. This trend was illustrated by consumers who said they have replaced fresh meat with pre-packaged cold cuts or fresh fish with tinned fish. Thus, the current study shows that shopping for groceries online encourages shoppers to repeatedly buy the same things, resulting in less variety in the products consumed. There is also a tendency to consume more processed foods given the challenges experienced by some shoppers to buy high-quality fresh produce online.

Jilcott Pitts et al.^(42)^ conducted a scoping review of the literature published between 2007 and 2017, aiming to identify the potential promises and pitfalls offered by OGS in relation to food and beverage purchases, including the impact of OGS on health. While the COVID-19 pandemic in 2019 and 2020 boosted online food purchasing, our study confirmed several of the findings of Jilcott Pitts et al.^(42)^ concerning OGS, such as the tendency to purchase less fresh produce and the aim to spend less by avoiding unplanned purchases, which may also have implications in terms of reducing household food waste^(52)^. We add new insights to their study by showing the considerable influence of product recommendation lists that encourage shoppers to repeatedly purchase the same items, resulting in less variety in the products consumed. We also found that for some consumers, there is a sense of security in regularly buying the same products. Additionally, there is a tendency to consume more processed foods due to the challenges of buying high-quality fresh produce online.

This research could encourage retailers to develop solutions that incite consumers to diversify their purchases and to buy more fresh produce. It is worth noting that similar criticisms were made when the first brick-and-mortar supermarkets opened in Europe in the 1960s and 1970s. For many years, fresh produce was neglected by retailers who were more interested in selling items that were less fragile and less subject to seasonal fluctuations^(53)^. However, retailers have made substantial progress over the years, and physical stores now offer a wide range of fresh produce. The same progress needs to be made with regard to fresh products sold online. Moreover, as the recommendation lists available to online consumers are currently based on their past purchasing behaviour, inciting them to select items similar to their previous orders, the suggestion system employed by grocery e-retailers could also be used to encourage customers to diversify their purchases. This would enhance e-service quality and potentially increase e-customer satisfaction while also improving online shoppers’ dietary balance.

While the current research offers valuable contributions, it does have some limitations. First, it was conducted in a single country (France). As researchers have shown that the factors influencing OGS can vary across cultures^(54,55)^, extending the investigation to different geographical contexts may broaden the validity of our findings. Second, the findings are derived from qualitative semi-structured interviews. To generalise the results, future research could be conducted using quantitative methods. Third, neuroscientific methods such as eye tracking are increasingly used in the context of retail research^(56)^. It could thus be beneficial to examine the way shoppers use online grocery websites using an eye tracker to provide a continuous objective measure of attention, allowing real-time observations to be collected^(57)^.

### Conclusion

As the first study conducted in France on this topic, the present paper provides further insights into the consequences of shopping online for food. Using in-depth, semi-structured individual interviews, the findings show that the transition from offline to online food shopping impacts the selection of products purchased, and consequently online shoppers’ dietary balance. As experts predict that grocery e-commerce penetration will double in the next 5 years, it is important to urge e-grocery retailers to develop solutions that encourage consumers to buy more fresh produce and sample a more varied diet. Additionally, our study demonstrates the importance of monitoring the influence of technology on the consumer buying process, especially with regard to food.

## Appendix A - Interview Guide

*The interview started with broader questions regarding the participant’s experience of shopping online for groceries*

When did you begin shopping online for groceries?

Do you always use the same e-retailer?

Was there a triggering factor to shopping online for groceries?

*The discussion then moved to more focused questions about the online consumer buying process:*

What is the share of your online and offline grocery purchases?

What are the different places where you typically shop for food, both online and offline?

What do you think about the product offerings online and offline?

Can you explain how you use a website or app to shop?

Have you used the product recommendation lists proposed by the website or app? If so, how?

How would you describe your planned and unplanned grocery purchases, both online and offline?

Have you noticed any changes in your diet since shopping online in terms of quantity (more of this, less of that) or quality (trying new products, excluding certain products)?

*The interview concluded with the following questions:*

Is there anything we haven’t discussed that you would like to add?

Do you have any questions for me?
